# Incidence of Brain Abnormalities Detected on Preoperative Brain MR Imaging and Their Effect on the Outcome of Cochlear Implantation in Children with Sensorineural Hearing Loss

**DOI:** 10.1155/2015/275786

**Published:** 2015-01-20

**Authors:** Xiao-Quan Xu, Fei-Yun Wu, Hao Hu, Guo-Yi Su, Jie Shen

**Affiliations:** Department of Radiology, The First Affiliated Hospital of Nanjing Medical University, No. 300, Guangzhou Road, Nanjing 210029, China

## Abstract

The incidence of sensorineural hearing loss (SNHL) increased gradually in the past decades. High-resolution computed tomography (HRCT) and magnetic resonance (MR) imaging, as an important part of preimplantation evaluation for children with SNHL, could provide the detailed information about the inner ear, the vestibulocochlear nerve, and the brain, so as to select suitable candidate for cochlear implantation (CI). Brain abnormalities were not rare in the brain MR imaging of SNHL children; however, its influence on the effect of CI has not been clarified. After retrospectively analyzing the CT and MR imaging of 157 children with SNHL that accepted preoperative evaluation from June 2011 to February 2013 in our hospital and following them during a period of 14.09 ± 5.08 months, we found that the white matter change, which might be associated with the history of medical condition, was the most common brain abnormality. Usually CI was still beneficial to the children with brain abnormalities, and the short-term hearing improvement could be achieved. Further study with more patients and longer follow-up time was needed to confirm our results.

## 1. Introduction

The incidence of sensorineural hearing loss (SNHL) was about 1/1000 in the newborns and about 9/1000 in the school age children, and it was still increasing gradually in the past decades [1]. Although previous studies indicated that the injury or infection during the parturition might be associated with the occurrence of SNHL, no definite pathogenesis was identified [2]. Nowadays, cochlear implantation (CI) has been accepted as the first-line treatment option for pediatric profound SNHL [3].

Imaging examination played an important role in the preimplantation evaluation of pediatric SNHL [4, 5]. High-resolution computed tomography (HRCT) and magnetic resonance (MR) imaging could provide the detailed information about the inner ear, the vestibulocochlear nerve, and the brain, which could help to select suitable candidate for CI and to predict whether CI would be beneficial or not.

Till now, several studies had clarified the incidence of the inner ear malformation or vestibulocochlear nerve deficiency and their influence on CI [6]. However, few studies focused on the effect of brain abnormalities detected on preoperative brain MR imaging on CI, although several studies had introduced its incidence.

Therefore, based on a large cohort of 157 patients who accepted preimplantation evaluation for SNHL in our hospital, we try to study the incidence of brain abnormalities and to clarify the influence of brain abnormalities on the hearing improvement after CI, further to clarify whether the brain abnormalities should be viewed as the contraindication of CI.

## 2. Material and Methods

### 2.1. Patients Group

This study was approved by our institutional review board. From June 2011 to February 2013, 157 children with SNHL accepted HRCT and MR scan as a part of preoperative evaluation. The 157 patients consisted of 89 boys and 68 girls with a mean age of 4.3 years (range: 1–15 years). Audiological evaluation indicated that there were 137 children with bilateral SNHL and 20 children with unilateral SNHL. Among all the 157 children with SNHL, 26 brain abnormalities were found on the brain MR imaging of 23 patients, and the incidence was 14.6% (23/157).

### 2.2. Data Acquisition

All patients underwent both HRCT and MR scans of the temporal bone. The HRCT scans were performed using a 16-slice spiral CT scanner (SOMATOM Emotion, Siemens, Germany). Parameters for the CT scanning are kV: 120, mAs: 28, and slice thickness: 1 mm. Volumetric acquisitions were reconstructed with 0.75 mm slice thickness throughout the temporal bone contiguously. The transverse imaging plane was parallel to the supraorbital-meatal line. This allowed creating multiplanar reconstructions in any plane. Bone window settings were designed as window level of +700 Hounsfield unit (HU) and window width of +4000 HU.

MR imaging was obtained on a 3.0 Tesla MR scanner (Siemens, Germany) with the matched eight-channel phased array coils. The MR protocol included axial T2-weighted imaging and axial T1-weighted imaging, as well as axial three-dimension sampling perfection with application optimized contrasts using different flip angle evolutions (3D-SPACE) imaging of temporal bones. Parameters for the T1 sequence are TR/TE, 250/2.5 ms; slice thickness, 5 mm. Parameters for the T2 sequence are TR/TE, 5000/100 ms; slice thickness, 5 mm. Parameters for the 3D-SPACE sequence are TR/TE, 1000/131 ms; flip angle, 120; averages, 2; field of view, 200 mm; matrix size, 384∗384; slice thickness, 0.5 mm; slice gap, 0 mm. The total scanning time was approximately 10 min for each patient. The 3D-SPACE sequences were reconstructed in the axial plane as well as in an oblique sagittal plane, approximately perpendicular to the long axis of each internal auditory canal (IAC) for viewing.

### 2.3. Image Assessment and Follow-Up

CT and MR imaging were assessed on a clinical picture archiving and communication system (PACS) workstation by two radiologists (Wu FY, Xu XQ). Brain abnormality was defined as any abnormal signal or structural abnormality demonstrated on the brain MR imaging. If the volume of the white matter lesion was less than 10% of that of the whole white matter, the lesion was categorized as a “focal lesion.” Otherwise, the lesion was classified as a “diffuse lesion.”

Patients were followed up mainly through the manner of phone calls. Except that one patient with bilateral SNHL was lost during the follow-up, we successfully followed up the other 22 patients with SNHL within a period of 14.09 ± 5.08 months (range: 7–27 months). Follow-up focused on evaluation of hearing improvement before and after CI. Children's parents were asked to finish the infant toddler-meaningful auditory integration scale (IT-MAIS) to evaluate the patients' ability to make meaningful use of sound before and after CI [7, 8].

IT-MAIS consisted of a series of 10 questions. Questions 1 and 2 related the bonding of the child to the device, including the willingness of the child to wear it and his ability to recognize and identify device malfunction. Questions 3 to 6 related the alerting to sound of the child when not in a “listening set.” Questions 7 to 10 related to the ability of the child to derive meaning from the auditory phenomena (Table 1). Response to each question was scored on a scale from 0 to 4, based on the frequency of reported behavior (0 = never, 1 = rarely, 2 = occasionally, 3 = frequently, and 4 = always). Therefore, a copy of IT-MAIS would score from 0 to 40. A score of 0 indicates the inability of the child to make use of sounds in his or her everyday environment, and a score of 40 indicates that the child can consistently understand and make use of sounds.

### 2.4. Statistical Analysis

The numerical data were averaged and reported as means ± standard deviation (SD). Improvement of IT-MALS score in “diffuse lesion” group and “focal lesion” group before and after CI was compared with Wilcoxon analysis. *P* < 0.05 was considered statistically significant. Statistical analysis was carried out with the SPSS 17.0 (Chicago, IL, USA).

## 3. Results

### 3.1. Incidence and Description of Brain Abnormalities

Among all the 157 patients with SNHL, 26 brain abnormalities were found on the MR imaging of 23 patients, and the incidence was 14.6% (23/157). The most common abnormality was pure white matter changes (*n* = 13), followed by pure structural abnormalities (*n* = 3), combination of white matter changes and structural abnormalities (*n* = 3), intraparenchymal cystic lesions (*n* = 3), and others (abnormal enlargement of subarachnoid space) (*n* = 1).

Among the 23 patients with brain abnormalities, concurrent abnormalities were found in 6 children, including bilateral mastoiditis (*n* = 3), malformed semicircular canal (*n* = 1), cochlear hypoplasia, absence of cochlear nerve (*n* = 1), and enlarged vestibular aqueduct (*n* = 1). The detailed demographic, clinical, and follow-up results of all the 23 patients were listed in Table 1.

### 3.2. Medical History during the Gestation and Perinatal Period

Among the 16 patients with white matter changes, 15 patients were successfully followed up. Among the 15 patients, 13 patients or their mothers had a history of medical condition that could be associated with the brain abnormalities, including viral influenza (*n* = 6), premature delivery (*n* = 4), cytomegalovirus (*n* = 3), rubella virus (*n* = 2), measles virus (*n* = 1), kernicterus (*n* = 1), and hypoxic ischemic encephalopathy (*n* = 1). The other SNHL patients had no history of medical condition that could be associated with the brain abnormalities.

### 3.3. Follow-Up of SNHL Children with Brain Abnormalities after CI

Twenty-three patients with brain abnormalities included 20 patients with bilateral SNHL and 3 patients with unilateral SNHL. After excluding the 3 unilateral cases that did not accept CI, one bilateral SNHL patient who gave up CI due to the complicated inner ear malformation, and one bilateral SNHL patient who was lost during the follow-up, a total of 18 patients with bilateral SNHL accepted CI.

**Figure 1:**
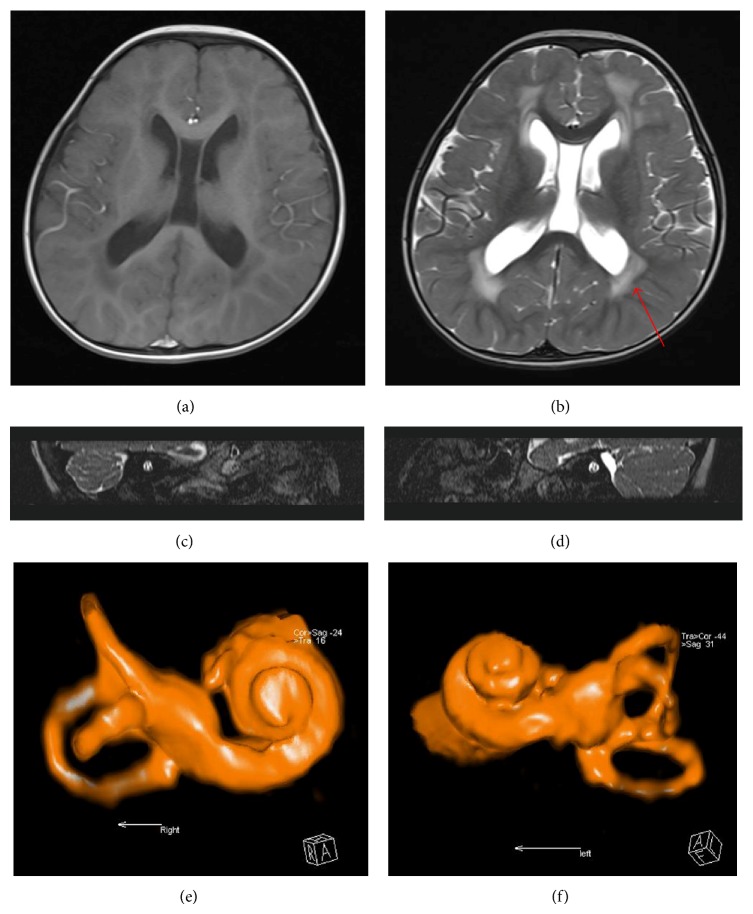
MR imaging of a representative patient with SNHL. (a, b) Axial T1 and T2 weighted imaging showed abnormal signal in the white matter in the bilateral periventricular area and enlargement of septum pellucidum. (c, d) Sagittal 3D-SPACE imaging showed the normal facial nerve, cochlear nerve, and vestibular nerves in the internal auditory canal. (e, f) Volume rendering imaging showed the normal structure of the inner ear. IT-MAIS score of this patient improved from 0 to 10 after CI.

Among the 18 patients, the IT-MAIS score improved from 1.55 ± 0.86 to 24.50 ± 5.68 after CI. Among the 18 patients, 11 patients with the white matter changes received CI, and the IT-MAIS score improved from 1.54 ± 0.82 to 24.55 ± 6.71. A representative case is shown in Figure 1.

Eleven patients with white matter changes included 5 patients with “diffuse lesion” and 6 patients with “focal lesion.” In the “diffuse lesions” group, except one patient who was 15 years old, the other 4 patients were 1, 1, 1, and 5 years old, respectively, with a mean age of 2.00 ± 2.00 years. In the “focal lesions” group, the mean age of the 6 patients was 2.17 ± 1.47 years. Meanwhile, in the “focal lesions” group, 2 patients had the concomitant abnormalities, including semicircular canal malformation in one patient and bilateral mastoiditis in another patient. In the “diffuse lesions” group, also 2 patients had the concomitant abnormalities, combined with bilateral mastoiditis in both of them. There were no other neurologic deficits or developmental delays associated with the brain abnormalities shown on the brain MR imaging in both of the two groups. In addition, there was no significant difference about the IT-MAIS score between two groups before CI (1.20 ± 1.41 versus 1.83 ± 0.71; *P* > 0.05). However, better hearing improvement was found in the “focal lesion” group than the “diffuse lesion” group after CI (27.00 ± 3.10 versus 18.20 ± 5.85; *P* < 0.05). Detailed IT-MAIS score change for children with SNHL before and after CI was showed in Table 2.

## 4. Discussion

The incidence of brain abnormalities in our study was 14.6%, relatively lower than previous studies, which demonstrated that the incidence of brain abnormalities varied from 20% to 56% [1, 9, 10]. The relative lower incidence might be explained that our hospital is the sole unit responsible for preoperative evaluation in our geographic region. The relative bigger base number of children with SNHL might reduce the incidence of brain abnormalities detected to a certain extent. Meanwhile, another study indicated that the brain abnormalities appeared more in the brain MR imaging of patients with bilateral rather than unilateral SNHL [11]. In our study, 23 SNHL patients with brain abnormalities included 20 bilateral and 3 unilateral cases, which is similar to the previous study.

The white matter changes were the most common brain abnormality. In our study, the incidence of white matter changes was 69.6%, which was similar to previous studies [1, 12]. The pathogenesis of white matter changes remains unclear, although several reports indicated that it might be related to previous insults, such as infection, ischemia, hypoxia, or prematurity [13]. In our study, 13 children or their mothers had an exact history of medical condition that could be associated with the white matter changes, including viral influenza in 6 patients, premature delivery in 4 patients, cytomegalovirus in 3 patients, rubella virus in 2 patients, measles virus in one patient, kernicterus in one patient, and hypoxic ischemic encephalopathy in one patient. No exact history of medical condition was found in the other 10 patients. Therefore, we speculated that the white matter changes might be partly associated with the history of medical condition during the gestation and perinatal period.

The white matter changes were viewed as an important marker of abnormal neurodevelopment and might help to predict potential future problems (seizure and intellectual impairment) in certain patients [14]. However, the impact of white matter changes on the hearing improvement after CI was still unclear. In our study, a total of 11 patients with the white matter changes accepted CI, and IT-MAIS score improved from 1.54 ± 0.82 to 24.55 ± 6.71 after CI. Therefore, we insisted that the SNHL patients with white matter changes could also acquire hearing improvement after CI. White matter changes should not be viewed as the absolute contraindication of CI. Meanwhile, we found that the patients in the “focal lesion” group could acquire probable better improvement than the patients in the “diffuse lesion” group (27.00 ± 3.10 versus 18.20 ± 5.85). In addition, as to the patients in whom SNHL was combined with other abnormalities, including arachnoid cyst or intraparenchymal cystic lesions, they also acquired the hearing improvement after CI. Therefore, these kinds of abnormalities should also not be viewed as the absolute contraindication of CI. However, due to the rarity of the SNHL patients with brain abnormalities and relatively short follow-up time, it might be difficult to make a definite conclusion regarding the impact of brain abnormalities on CI. Further study with more patients and longer follow-up time was needed to confirm our results.

Our study had several limitations. First, our study was a retrospectively clinical observational and descriptive study. We could just describe the different brain abnormalities existing in brain MR imaging of the patients with SNHL and correlated the imaging findings with the medical histories, so as to analyze the potential cause of the brain abnormalities. However, no exact pathological evidence could be achieved for supporting our research result. Secondly, the number of the SNHL patients with brain abnormalities and the period of follow-up time were limited. Further study with more patients and longer follow-up time was needed to confirm our results.

## 5. Conclusion

Brain abnormalities were common on the brain MR imaging of the patients with SNHL; therefore, the whole brain MR scan was necessary in the preimplantation evaluation. The white matter changes, which were the most common brain abnormalities, might be associated with the history of medical condition during the gestation and perinatal period. Brain abnormalities might not influence the short-term hearing improvement after CI. Further study with more patients and longer follow-up time was needed to confirm our results.

## Figures and Tables

**Table 1:** Follow-up of 23 SNHL patients with brain abnormalities after cochlear implantation.

Abnormal brain MRI findings	Concurrent abnormalities	Y/S	U/B	CI	IT-MAIS		FU (Mon)	Focal/diffuse
					Before	After		
*White matter changes *								
Bilateral frontal and temporal subcortical area Bilateral periventricular area Ventriculomegaly	—	1/M	B	Yes	0	10	15	Diffuse
Multiple WMC lesions	—	1/M	U	No	—	—	12	—
Right posterior horn of lateral ventricle	—	4/M	B	Yes	3	31	14	Focal
Bilateral posterior horn of lateral ventricle	Malformed semicircular canal	2/F	B	Yes	1	34	16	Focal
Bilateral periventricular area Bilateral centrum semiovale	—	1/M	U	No	—	—	9	—
Bilateral frontal and parietal subcortical area	—	1/M	B	Yes	1	29	27	Focal
Bilateral posterior horn of lateral ventricle	Bilateral mastoiditis	1/M	B	Yes	2	27	18	Focal
Multiple WMC lesions	—	1/F	B	Yes	1	23	12	Diffuse
Multiple WMC lesions Arachnoid cyst (left temporal area)	—	15/F	B	Yes	2	16	20	Diffuse
Multiple WMC lesions	Bilateral mastoiditis	1/M	B	Yes	2	24	8	Diffuse
Bilateral periventricular area	—	1/M	B	Yes	2	25	7	Focal
Bilateral frontal subcortical area Bilateral posterior horn of lateral ventricle Arachnoid cyst (left temporal area)	Bilateral mastoiditis	5/M	B	Yes	1	24	11	Diffuse
Bilateral periventricular area	—	3/M	U	No	—	—	18	—
Right posterior horn of lateral ventricle	—	4/F	B	Yes	2	27	18	Focal
Bilateral posterior horn of lateral ventricle Bilateral centrum semiovale	Cochlear hypoplasia Absence of cochlear nerve	3/F	B	No	—	—	11	—
Bilateral periventricular area	—	1/M	B	L^*^	—	—	—	—
*Structural abnormalities *								
Cisterna magna enlargement	—	2/M	B	Yes	1	28	15	—
Arachnoid cyst (left temporal area)	—	1/M	B	Yes	1	24	12	—
Arachnoid cyst (left temporal area)	—	3/F	B	Yes	2	29	9	—
*Intraparenchymal cystic lesions *								
Intraparenchymal cystic lesions (left lateral ventricular trigone)	—	1/M	B	Yes	2	22	7	—
Intraparenchymal cystic lesions (right occipital area)	—	3/F	B	Yes	3	25	22	—
Intraparenchymal cystic lesions (right centrum semiovale)	—	3/M	B	Yes	2	26	13	—
*Others *								
Abnormal enlargement of subarachnoid space (bilateral frontal and temporal area)	Enlarged vestibular aqueduct	1/F	B	Yes	0	17	16	—

Y, year old; S, sex; U, unilateral SNHL; B, bilateral SNHL; CI, cochlear implantation; FU, follow-up.

L^*^ means the patient was lost during the follow-up.

**Table 2:** IT-MAIS score of children with SNHL before and after cochlear implantation.

Group	All SNHL children (*n* = 18)		WMC group (*n* = 11)		WMC group (*n* = 11)	
IT-MAIS score	Before	After	Before	After	Diffuse (*n* = 5)	Focal (*n* = 6)
	1.55 ± 0.86	24.50 ± 5.68	1.54 ± 0.82	24.55 ± 6.71	18.20 ± 5.85^a^	27.00 ± 3.10^b^
*P*	*P* < 0.05		*P* < 0.05		*P* < 0.05	

WMC indicates white mater change. The number in the parenthesis means the number of the patients in each group. ^a,b^The score means the improvement of IT-MAIS score in the two groups.
